# Supplementary material for: Efficacy of drug treatment for severe melioidosis and eradication treatment of melioidosis: A systematic review and network meta-analysis
Source: PLoS Negl Trop Dis. 2023 Jun 12;17(6):e0011382. doi: 10.1371/journal.pntd.0011382 (PMC10289671; doi:10.1371/journal.pntd.0011382)
Supplement: S4 Table — (DOCX) [file pntd.0011382.s004.docx]

**S4 Table**. Treatment comparisons and data used for network meta-analysis of eradication therapy (adverse drug events)

| **Author** | **Year** | **Intervention** | **Recurrence** | **Non-recurrence** |
| --- | --- | --- | --- | --- |
| Anunnatsiri et al. [1] | 2020 | TMP-SMX 12 week | 23 | 278 |
| Anunnatsiri et al. [1] | 2020 | TMP-SMX 20 week | 45 | 276 |
| Chaowagul et al. [2] | 2005 | TMP-SMX+doxycycline+chloramphenicol >12 week | 37 | 54 |
| Chaowagul et al. [2] | 2005 | TMP-SMX+doxycycline >12 week | 20 | 69 |
| Chaowagul et al. [3] | 1999 | TMP-SMX+Doxycycline+chloramphenicol >12 week | 14 | 30 |
| Chaowagul et al. [3] | 1999 | Doxycycline 20 weeks | 9 | 34 |
| Chetchotisakd et al. [4] | 2001 | Ciprofloxacin+azithromycine 12 weeks | 3 | 29 |
| Chetchotisakd et al. [4] | 2001 | TMP-SMX+doxycycline >12 week | 4 | 29 |
| Chetchotisakd et al. [5] | 2014 | TMP-SMX 20 weeks | 122 | 189 |
| Chetchotisakd et al. [5] | 2014 | TMP-SMX+doxycycline >12 weeks | 167 | 148 |

**References**

1. Anunnatsiri S, Chaowagul W, Teparrukkul P, Chetchotisakd P, Tanwisaid K, Khemla S, et al. A Comparison Between 12 Versus 20 Weeks of Trimethoprim-sulfamethoxazole as Oral Eradication Treatment for Melioidosis: An Open-label, Pragmatic, Multicenter, Non-inferiority, Randomized Controlled Trial. Clin Infect Dis. 2021;73(11):e3627-e33. Epub 2020/07/30. doi: 10.1093/cid/ciaa1084. PubMed PMID: 32725199; PubMed Central PMCID: PMCPMC8662794.

2. Chaowagul W, Chierakul W, Simpson AJ, Short JM, Stepniewska K, Maharjan B, et al. Open-label randomized trial of oral trimethoprim-sulfamethoxazole, doxycycline, and chloramphenicol compared with trimethoprim-sulfamethoxazole and doxycycline for maintenance therapy of melioidosis. Antimicrob Agents Chemother. 2005;49(10):4020-5. Epub 2005/09/29. doi: 10.1128/aac.49.10.4020-4025.2005. PubMed PMID: 16189075; PubMed Central PMCID: PMCPMC1251512.

3. Chaowagul W, Simpson AJ, Suputtamongkol Y, Smith MD, Angus BJ, White NJ. A comparison of chloramphenicol, trimethoprim-sulfamethoxazole, and doxycycline with doxycycline alone as maintenance therapy for melioidosis. Clin Infect Dis. 1999;29(2):375-80. Epub 1999/09/07. doi: 10.1086/520218. PubMed PMID: 10476745.

4. Chetchotisakd P, Chaowagul W, Mootsikapun P, Budhsarawong D, Thinkamrop B. Maintenance therapy of melioidosis with ciprofloxacin plus azithromycin compared with cotrimoxazole plus doxycycline. Am J Trop Med Hyg. 2001;64(1-2):24-7. Epub 2001/06/27. doi: 10.4269/ajtmh.2001.64.24. PubMed PMID: 11425157.

5. Chetchotisakd P, Chierakul W, Chaowagul W, Anunnatsiri S, Phimda K, Mootsikapun P, et al. Trimethoprim-sulfamethoxazole versus trimethoprim-sulfamethoxazole plus doxycycline as oral eradicative treatment for melioidosis (MERTH): a multicentre, double-blind, non-inferiority, randomised controlled trial. Lancet. 2014;383(9919):807-14. Epub 2013/11/29. doi: 10.1016/s0140-6736(13)61951-0. PubMed PMID: 24284287; PubMed Central PMCID: PMCPMC3939931.
