# Supplementary material for: Efficacy of drug treatment for severe melioidosis and eradication treatment of melioidosis: A systematic review and network meta-analysis
Source: PLoS Negl Trop Dis. 2023 Jun 12;17(6):e0011382. doi: 10.1371/journal.pntd.0011382 (PMC10289671; doi:10.1371/journal.pntd.0011382)
Supplement: S1 Fig — (DOCX) [file pntd.0011382.s005.docx]

**S1 Fig.** Risk of bias assessment for treatment of severe melioidosis

**References**

1. Cheng AC, Limmathurotsakul D, Chierakul W, Getchalarat N, Wuthiekanun V, Stephens DP, et al. A randomized controlled trial of granulocyte colony-stimulating factor for the treatment of severe sepsis due to melioidosis in Thailand. Clin Infect Dis. 2007;45(3):308-14. Epub 2007/06/30. doi: 10.1086/519261. PubMed PMID: 17599307.

2. Chetchotisakd P, Porramatikul S, Mootsikapun P, Anunnatsiri S, Thinkhamrop B. Randomized, double-blind, controlled study of cefoperazone-sulbactam plus cotrimoxazole versus ceftazidime plus cotrimoxazole for the treatment of severe melioidosis. Clin Infect Dis. 2001;33(1):29-34. Epub 2001/06/05. doi: 10.1086/320878. PubMed PMID: 11389491.

3. Chierakul W, Anunnatsiri S, Short JM, Maharjan B, Mootsikapun P, Simpson AJ, et al. Two randomized controlled trials of ceftazidime alone versus ceftazidime in combination with trimethoprim-sulfamethoxazole for the treatment of severe melioidosis. Clin Infect Dis. 2005;41(8):1105-13. Epub 2005/09/16. doi: 10.1086/444456. PubMed PMID: 16163628.

4. Simpson AJ, Suputtamongkol Y, Smith MD, Angus BJ, Rajanuwong A, Wuthiekanun V, et al. Comparison of imipenem and ceftazidime as therapy for severe melioidosis. Clin Infect Dis. 1999;29(2):381-7. Epub 1999/09/07. doi: 10.1086/520219. PubMed PMID: 10476746.

5. Sookpranee M, Boonma P, Susaengrat W, Bhuripanyo K, Punyagupta S. Multicenter prospective randomized trial comparing ceftazidime plus co-trimoxazole with chloramphenicol plus doxycycline and co-trimoxazole for treatment of severe melioidosis. Antimicrob Agents Chemother. 1992;36(1):158-62. Epub 1992/01/01. doi: 10.1128/aac.36.1.158. PubMed PMID: 1590682; PubMed Central PMCID: PMCPMC189245.

6. Suputtamongkol Y, Rajchanuwong A, Chaowagul W, Dance DA, Smith MD, Wuthiekanun V, et al. Ceftazidime vs. amoxicillin/clavulanate in the treatment of severe melioidosis. Clin Infect Dis. 1994;19(5):846-53. Epub 1994/11/01. doi: 10.1093/clinids/19.5.846. PubMed PMID: 7893868.

7. Thamprajamchit S, Chetchotisakd P, Thinkhamrop B. Cefoperazone/sulbactam + co-trimoxazole vs ceftazidime + co-trimoxazole in the treatment of severe melioidosis: a randomized, double-blind, controlled study. J Med Assoc Thai. 1998;81(4):265-71. Epub 1998/06/12. PubMed PMID: 9623020.

8. White NJ, Dance DA, Chaowagul W, Wattanagoon Y, Wuthiekanun V, Pitakwatchara N. Halving of mortality of severe melioidosis by ceftazidime. Lancet. 1989;2(8665):697-701. Epub 1989/09/23. doi: 10.1016/s0140-6736(89)90768-x. PubMed PMID: 2570956.
