# Supplementary material for: Efficacy of drug treatment for severe melioidosis and eradication treatment of melioidosis: A systematic review and network meta-analysis
Source: PLoS Negl Trop Dis. 2023 Jun 12;17(6):e0011382. doi: 10.1371/journal.pntd.0011382 (PMC10289671; doi:10.1371/journal.pntd.0011382)
Supplement: S3 Fig — (DOCX) [file pntd.0011382.s007.docx]

**S3 Fig.** Network map of hospital mortality for treatment of severe melioidosis.

The size of each node shows the number of participants that were randomly assigned to a treatment comparison. The width of the lines shows the number of comparative studies between the two treatments.
