# Supplementary material for: Efficacy of drug treatment for severe melioidosis and eradication treatment of melioidosis: A systematic review and network meta-analysis
Source: PLoS Negl Trop Dis. 2023 Jun 12;17(6):e0011382. doi: 10.1371/journal.pntd.0011382 (PMC10289671; doi:10.1371/journal.pntd.0011382)
Supplement: S10 Fig — (DOCX) [file pntd.0011382.s014.docx]

**S10 Fig.** Comparison adjusted funnel plot for the outcome of drug discontinuation
