# Supplementary material for: Efficacy of drug treatment for severe melioidosis and eradication treatment of melioidosis: A systematic review and network meta-analysis
Source: PLoS Negl Trop Dis. 2023 Jun 12;17(6):e0011382. doi: 10.1371/journal.pntd.0011382 (PMC10289671; doi:10.1371/journal.pntd.0011382)
Supplement: Attachment — Submitted filename: MelioRx-PeerRVcomments.pdf [file pntd.0011382.s018.pdf]

Efficacy of drug treatment for severe melioidosis and eradication treatment of melioidosis: A systematic review and network meta-analysis.

Reviewer overview:

This is an interesting analysis of drug therapy for melioidosis where perhaps good quality data is lacking. It is generally well written. The use of a "A two stage NMA with surface under the cumulative ranking curve (SUCRA) was used to estimate the comparative efficacy of treatment regimens" is interesting and may be unfamiliar to many readers, including myself as reviewer.

It seems there is some inference with this method, especially were no direct comparison is made in the included studies.

If methods employed are sound (as assessed by someone knowledgeable in NMA) then I think this study has utility of suggesting further assessment of using adjunct G-CSF in severe melioidosis therapy and further well-planned prospective studies to assess the best eradication therapy.

I have no major concerns (assuming the analysis is performed correctly and is a valid application) but please see general comments, along with minor comments for revision.

General comments:

All the studies were from Thailand (line 246), this seems a bit unusual given your search strategy did not specify studies had to be a certain geographic area? Any comments on this and how it might affect your results/ implications/ conclusions? Given specific treatment modalities / local Thai strains or other factors.

What are the current antibiotic guidelines for treating melioidosis in Thailand and do these differ from other country guidelines? Is G-CSF recommended in any Thailand treatment guidelines for melioidosis?

Much of the discussion seems to focus a lot on comparison to Chloramphenicol/Doxy/TMP-STX to CAZ + G-CSF, however this seems a bit misplaced as this "triple therapy" should be rarely used now and is not really standard of care – I think the focus should be more on is CAZ + G-CSF better then monotherapy with CAZ or a carbapenem. In general, as a clinician many of the comparison are not overly useful.

Is it worth outlining a bit more what SUCRA values actually mean to people who are not familiar with NMA? Its easier for most readers to understand what RR are whether this is significant or not – however much of your results rely on the SUCRA values, when for example CAZ vs CAZ + G-CSF is not significant, RR 0.81 (0.61-1.06). But then your SUCRA for CAZ + G-CSF is 79.7% (the highest) and therefore the conclusion is this is most likely the best therapy. I think it could be discussed contextualised a bit better how you have come to this conclusion.

Minor:

Line 31: Full stop/ period after regimens.

Line 50: Many guidelines suggest meropenem, any reason to state imipenem instead?

Line 70: Small distinction, but it is the U.S. Centers [with an s] of Disease…

Line 74: Suggest delete “continuously”

Line 76: “But the death rate doubly increases when patients have septicemia”- rewrite without the phase “doubly increases” Note also ref 5 has had a retraction due to significant issues with the article – consider not using this reference. An issue I noted, for example, was was that the 21% mortality rate stated (in Thailand) had a reference to a case report of glanders in a human, or is not referenced when this same rate is stated to be the mortality rate in Australia.

Line 77: Carbapenem is not an antibiotic as such, rather a class of antibiotics

Line 78-79: “Mortality rate was still high (37%), especially in patients with septicemia” This might need a bit more context as you state antibiotics reduced mortality rate but in a previous sentence say the mortality rate is approx 41% with reference to another study. Is this comparing untreated/ undertreated to optimally treated etc?

Line 83-84: “Unfortunately, none of these studies demonstrated significant benefit of these treatment regimens over ceftazidime to reduce mortality in severe melioidosis patients”- were all these studies comparing a treatment regimen to ceftazidime?

Line 132-134: Often the reviewers who did the review for selection and the 3rd party consensus would have this defined by bracketed initials.

Line 154: What do you mean by “clinical-confirmed” recurrence?

Line 155: Typo- Clulture

Line 164: As above which reviewer initials?

Line 164-166: “Outcomes of interest including frequency of patients who developed or did not develop the outcomes for each treatment were also extracted”- rewrite.

Line 176-178: As per prior comments consider specifying which reviewers did what.

Line 201-202 and Fig 1, line 229-235: It is a bit confusing from reading the text and comparing to this figure- did you have 8 or 9 studies that underwent analysis for treatment of severe melioidosis?

Line 247-249: Make it clear you are also not just looking at a catch all “co-morbid conditions” “The rates of the co-morbid conditions of diabetes mellitus and chronic kidney disease ranged..”

Line 250: Keep consistent decimal points.

Line 260, Tab 1 Sookpranee, 1992: Pneumonia box is empty.

Line 267-273: “Treating with chloramphenicol plus doxycycline plus TMP-SMX had a significantly greater risk of hospital mortality than other treatments except for cefoperazone-salbactam plus TMP-SMX, with RRs of 2.22 (95% CI: 1.28-3.86) for ceftazidime plus TMP-SMX, 1.96 (95% CI: 1.13-3.38) for amoxicillin-clavulanic acid, 2.01 (95% CI: 1.12-3.59) for Imipenem, 2.38 (95% CI:1.39-4.07) for ceftazidime plus G-CSF, and 1.92 (95% CI: 1.21-3.05) for ceftazidime. Ceftazidime plus G-CSF had a lower hospital mortality rate than all other treatments, although the results were not statistically significant”

Where did you get the RR of 2.22 from- this is not in the table?
I take it you single out the treatment regimen of chloramphenicol/ doxycycline/ TMP-SMX because this had the most significant results compared to other treatments but is this clinically relevant – is this regimen used much in practice? It might be worth highlighting some of the RR, even if not significant for other treatment regimens that are often used.

Line 280-297, Tab 2: A somewhat busy and hard to interpret table. This might be my unfamiliarity with NMA, but clearly comparisons are made between treatment regimens such as imipenem vs co-amoxyclav or TMP-STX + doxy + chloramphenicol vs imipenem (which is a significant result). Is might be worth outlining which results are "derived" or indirect vs from direct comparison from studies (which would seem to hold more weight).

Line 293: "disseminated disease"- how is this defined? Compared to bacteraemia.

Line 318-323, Tab 4: Similar to comments on table 2. Consider outlining direct comparison vs NMA derived values.

Line 371-376 Tab 5, comments per other tables.

Line 403-404: "Currently, ceftazidime with or without TMP-SMX is the preferred first line treatment for severe melioidosis"- reference any relevant guidelines. Define specifically when addition of TMP-SMX is thought to be of benefit – i.e. patients with focal disease rather than just pneumonia/ bacteraemia.

Line 478-479: "Inclusion of more data from more studies would strengthen our conclusion". Well, it might not – I think you need to highlight that your findings are perhaps more hypothesis generating, far from good quality evidence to suggest G-CSF should definitively be utilised and that we need well designed prospective studies looking into therapy for melioidosis. I think the fact that direct meta-analysis could not be performed on "standard therapies" for melioidosis and NMA had to be performed – with many results lacking power / not reaching significance- really highlights the need for good quality clinical trials to improve treatment of melioidosis.
