# Supplementary material for: Efficacy of drug treatment for severe melioidosis and eradication treatment of melioidosis: A systematic review and network meta-analysis
Source: PLoS Negl Trop Dis. 2023 Jun 12;17(6):e0011382. doi: 10.1371/journal.pntd.0011382 (PMC10289671; doi:10.1371/journal.pntd.0011382)
Supplement: Attachment — Submitted filename: Response to reviewers comments_23_Mar_2023.docx [file pntd.0011382.s019.docx]

**Response to reviewer’s comments**

<b>Key Review Criteria Required for Acceptance?</b></br></br> As you describe the new analyses required for acceptance, please consider the following:</br></br>

<b>Methods</b></br></br>

-Are the objectives of the study clearly articulated with a clear testable hypothesis stated?</br> -Is the study design appropriate to address the stated objectives?</br> -Is the population clearly described and appropriate for the hypothesis being tested?</br> -Is the sample size sufficient to ensure adequate power to address the hypothesis being tested?</br> -Were correct statistical analysis used to support conclusions?</br> -Are there concerns about ethical or regulatory requirements being met?</br></br>

**Reviewer #1:** -

**Reviewer #2:** Yes but see my comments below

**Reviewer #3:** I can not comment in detail of the methods of the technique of NMA used in this submission. The authors seem to have followed PRISMA guidelines and acknowledge limitations in the methods.

--------------------

<b>Results</b></br></br>

-Does the analysis presented match the analysis plan?</br> -Are the results clearly and completely presented?</br> -Are the figures (Tables, Images) of sufficient quality for clarity?</br></br>

**Reviewer #1: -**

**Reviewer #2:** See comments below

**Reviewer #3:** I think results are presented reasonably and it is discussed that many of the results do not reach significance or are under-powered. Some of the results were focused a bit on treatment regimens that have little current relevance.

**Response:** Because the goal of this systematic review is to thoroughly review and compare the efficacy of all available treatments for severe melioidosis and eradication therapies of melioidosis. Therefore, we did not restrict the time period for searching and selecting the eligible studies. The old treatment regimens were commonly compared with ceftazidime, which is the most common comparator in almost all studies assessing the treatment of melioidosis (please see the figure below). Therefore, combining these studies with the studies that assessed the new treatment regimens can increase the sample size and then increase the power to detect the difference. Furthermore, ceftazidime data from old treatment regimen studies could be used as a common comparator to estimate indirect treatment effect in network meta-analysis (please see network map figure below).

*Network map of hospital mortality for treatment of severe melioidosis*

--------------------

<b>Conclusions</b></br></br>

-Are the conclusions supported by the data presented?</br> -Are the limitations of analysis clearly described?</br> -Do the authors discuss how these data can be helpful to advance our understanding of the topic under study?</br> -Is public health relevance addressed?</br></br>

**Reviewer #1:** The treatment of melioidosis remains a significant issue in endemic areas. This study provides valuable data from numerous landmark trials for clinicians to select the most suitable regimens for their patients. The thoroughness of the data is a strength of the study. However, there are a few concerns regarding the discussion section.

1. Each study included in the paper was conducted to address a specific question. For example, Anunnatsiri et al (2020) concluded that patients with melioidosis who had no residual foci of infection after 12 weeks of oral co-trimoxazole could safely discontinue antibiotics. Chetchotisakd et al (2014) found that adding doxycycline to co-trimoxazole did not provide any additional benefit for maintenance therapy compared to co-trimoxazole monotherapy. Pooling the data across studies may lead to a misleading conclusion for clinicians.

**Response:** Thank you very much for your comment. We all agreed that each included study addressed each specific question and took into account the various treatment interventions. However, the primary goals of all included studies were similar: to assess the efficacy of eradication therapies in melioidosis patients following acute treatment. Therefore, there is a rationale for pooling the data across the studies using the network meta-analysis. Applying network meta-analysis (NMA) can compare three or more interventions simultaneously in a single analysis by combining both direct and indirect evidence across all included studies. The advantages of NMA are that it can estimate the relative treatment effects of any pair of interventions in the network and usually yields more precise estimates than a single direct or indirect estimate. In addition, NMA can estimate the ranking and hierarchy of interventions. Therefore, we applied the NMA to pool the data across the included studies to compare the efficacy among all available treatments for melioidosis. A valid network meta-analysis, however, is based on the assumption that the different sets of studies included in the analysis are, on average, comparable in terms of all significant characteristics that may influence the relative treatment effects, such as study participants' characteristics. In our analysis, the inclusion criteria for the study’s participants and outcomes are similar among all included studies. However, some factors such as time periods when conducting the studies are different and may affect the validity of our analysis. We have discussed and addressed this limitation in the discussion part (please see page 29, lines 543-558).

2. Each study was conducted at a specific time period (from 1989 to 2020). Over the course of more than 30 years, there have been significant improvements in medical care, particularly in supportive treatment in critical care units, which have played a major role in lowering mortality rates. The author asserts that granulocyte-colony stimulating factor (G-CSF) is the most efficacious treatment for severe melioidosis, but this may not be the case in present times. Due to the latest sepsis campaign, G-CSF is not recommended for the treatment of sepsis and septic shock. The latest randomized controlled trial of G-CSF for melioidosis was published in 2007, and it is unclear whether its benefit is still applicable.

**Response:** Thank you very much for your comments. We are also concerned about the long time period of the included studies, which makes the quality of supportive treatment dissimilar among the included studies. We have addressed this issue in the limitation (see page 29, lines 552–558). For the concern about G-CSF, we have toned down our conclusions in the main text (page 25, lines 444-447 and page 29, lines 562-570), abstract (page 2, lines 44-45) and author summary (page 4, lines 65-67). The findings from more recent evidence of G-CSF have been added to the discussion part (please see page 26, lines 469-473).

**Reviewer #2:** See comments below

**Reviewer #3:** Mostly- but I think the limitations and context of having to use NMA for the analysis could have maybe been better contextualized or discussed in more detail to a general audience who may not be to familiar with the statistics and why the authors used these methods.

**Response:** The advantages of NMA and the rationale of applying NMA in our study have been added to the introduction part (please see page 7, lines 128-134). The limitations of NMA have been discussed in the limitation part (please see pages 29, lines 543-558).

--------------------

<b>Editorial and Data Presentation Modifications?</b></br><br/> Use this section for editorial suggestions as well as relatively minor modifications of existing data that would enhance clarity. If the only modifications needed are minor and/or editorial, you may wish to recommend “Minor Revision” or “Accept”.

**Reviewer #1: -**

**Reviewer #2:** Some minor editing of English required

**Response:** The English language has been edited by native speaker.

**Reviewer #3:** Minor Revision.

--------------------

<b>Summary and General Comments</b></br></br> Use this section to provide overall comments, discuss strengths/weaknesses of the study, novelty, significance, general execution and scholarship. You may also include additional comments for the author, including concerns about dual publication, research ethics, or publication ethics. If requesting major revision, please articulate the new experiments that are needed.

**Reviewer #1:** In my opinion, the author should avoid concluding which option is the most efficacious treatment and instead provide a narrative discussion, highlighting the two limitations mentioned above in the text.

**Response.** We have revised the conclusion in the abstract (page 2, lines 44-45), author summary (page 4, lines 65-67), and main text (page 25, lines 444-447 and page 29, lines 562-570) as suggested. The two limitations have been mentioned in the discussion part (please see page 26, lines 469-473 and see page 29, lines 552–558).

**Reviewer #2:** This paper describes a network meta-analysis of treatments for melioidosis based on an analysis of 9 studies of intensive phase treatment and 6 of oral eradication treatment.

1. My major concern is that the conclusions, particularly those relating to intensive phase treatment, are presented in the summary in such a way as to have a potentially major impact on the way melioidosis is treated without sufficient supporting evidence. The statement “ceftazidime plus G-CSF was the most efficacious treatment for severe melioidosis” implies that anything else might represent sub-optimal treatment, whereas detailed reading of the paper makes it clear that the difference was not statistically significant and that “further multi-center RCTs with larger sample sizes are required to confirm the benefit of G-CSF in patients with severe melioidosis”. In addition, G-CSF may well not be practical or affordable (or indeed necessary) for routine use in many melioidosis-endemic areas. I would suggest that this statement should not be made anywhere in the paper without relevant caveats, such as the statements in the discussion that “further multi-center RCTs with larger sample sizes are required to confirm the benefit of G-CSF in patients with severe melioidosis” and “the benefit of G-CSF may be greater in patients with lower severity of disease”.

**Response:** We have revised the conclusions in the abstract (page 2, lines 44-50), author summary (page 4, lines 65-67), and main text (page 25, lines 444-447 and page 29, lines 562-570) as suggested.

2. I am also concerned that the conclusion that ceftazidime plus TMP-SMX was the second most effective treatment for decreasing hospital mortality in patients with severe melioidosis might encourage those treating melioidosis patients to use TMP-SMX routinely, thereby exposing many patients to unnecessary risks of adverse effects from TMP-SMX. I think that the approach advocated by the Darwin group, whereby TMP-SMX is only added in a selected sub-group of patients (Sullivan RP, Marshall CS, Anstey NM, Ward L, Currie BJ. 2020 Review and revision of the 2015 Darwin melioidosis treatment guideline; paradigm drift not shift. PLoS neglected tropical diseases. 2020;14(9):e0008659), is more appropriate.

**Response:** We revised the conclusions based on your suggestion. In addition, the cautions of using TMP-SMX with ceftazidime have been discussed and added to the discussion part according to your comments (please see page 26, lines 480-486).

3. Secondly, whilst I recognise that the methodology of this sort of analysis requires that only RCTs can be considered in the meta-analysis, this automatically means that none of the extensive experience from Australia, where the number of cases of melioidosis seen is insufficient to enable RCTs to be undertaken, can be taken into account. In fact recent experience in Darwin has shown that their current guidelines (see above) have achieved a reduction in overall mortality to less than 10% (Currie BJ, Mayo M, Ward LM, Kaestli M, Meumann EM, Webb JR, et al. The Darwin Prospective Melioidosis Study: a 30-year prospective, observational investigation. The Lancet Infectious diseases. 2021;21(12):1737-46). Such guidelines are able to take into account the considerable variations between patients in the sites and extent of infection which it would be almost impossible to do in an RCT. At the very least this might warrant mention in the discussion. This would also enable discussion of the judicious and selective use of carbapenems as discussed in this and other papers from Australia. It is unfortunate that an RCT comparing ceftazidime and meropenem was started in Thailand many years ago (see <https://clinicaltrials.gov/ct2/show/NCT00579956>) but never finished due to the withdrawal of support by the pharmaceutical company, and this too may be worthy of mention.

**Response:** The findings from the Darwin Prospective Melioidosis study, the judicious and selective use of carbapenem described in this study, and the withdrawal of a RCT comparing ceftazidime and meropenem have been mentioned and discussed in the discussion part, as you suggested (please see page 27, lines 494-507). In addition, we have revised the paragraph mentioned about the mortality rate in melioidosis patients in the introduction by adding the information from this study (please see page 5, lines 85-88).

4. It might also be worth mentioning in the introduction that the last Cochrane review on melioidosis was conducted in 2002 (<https://www.cochranelibrary.com/cdsr/doi/10.1002/14651858.CD001263/full?highlightAbstract=melioidosi%7Cmelioidosis>).

**Response:** The findings from this Cochrane review have been mentioned in the introduction (please see page 6, lines 119-127).

5. The finding that TMP-SMX for 20 weeks had the lowest probability of drug discontinuation, even when compared with TMP-SMX for 12 weeks, is counter-intuitive and warrants some discussion.

**Response:** This issue has been mentioned and discussed in the discussion part (please see page 28, lines 521-527).

**Minor Comments**

1. Line 50. Ceftazidime misspelled.

**Response:** Corrected (see page 4, line 55).

2. Line 51. See my comment above about mortality, which over the past 5 years has been only 6%.

**Response:** This paragraph has been revised (please see page 4, lines 56).

3. Line 52. Change ‘factors’ to ‘factor’ and add ‘or’ before trimethoprim-sulfamethoxazole.

**Response:** Changed (see page 4, line 57).

4. Line 53. Change ‘mono-antibiotics’ to ‘antibiotic monotherapy’.

**Response:** Changed (see page 4, line 58).

5. Line 71. These statements should be referenced. I suspect that the authors are using the 2004 version of the WHO Laboratory Biosafety Manual (3rd edition) which has now been superseded.

**Response:** The 4^th^ edition of Laboratory biosafety manual from the World Health Organization has been cited for the reference of these statements (please see page 5, line 81).

6. Lines 75-76. Reference 5 has actually been retracted as it contained some grossly misleading material (see <https://pubmed.ncbi.nlm.nih.gov/32609720/>). Furthermore, it is actually very misleading to quote a single mortality rate (21%) as this varies hugely in place and time - the most recent estimate in Australia is less than 10% - see my comments above.

**Response:** Instead of quoting a single value, this paragraph has been revised to state a range of mortality rates. Moreover, reference no. 5 has been deleted according to your comments (please see page 5, lines 85-88).

7. Lines 89-90. Again, it is very misleading to quote a single value (23%) for recurrence rates as it is hugely variable and dependent on a number of factors.

**Response:** This sentence has been revised by stating a range of recurrent rate instead of quoting a single value (please see page 6, lines 102-103).

8. Lines 92-4. This statement requires a reference.

**Response:** We have cited the reference for this sentence (please see page 6, line 107).

***Reference:***

Ketheesan N, editor. Melioidosis: A Century of Observation and Research. Amsterdam: Elsevier B.V.; 2012.

9. Line 282. Add ‘with’ after ‘compared’.

**Response:** We have added ‘with’ after ‘compared’ as suggested (please see page 15, line 323).

10. Line 367. Review punctuation in this sentence.

**Response:** Corrected (please see page 22, line 414).

**Reviewer #3:** Efficacy of drug treatment for severe melioidosis and eradication treatment of melioidosis: A systematic review and network meta-analysis.

Reviewer overview:

This is an interesting analysis of drug therapy for melioidosis where perhaps good quality data is lacking. It is generally well written. The use of a “A two stage NMA with surface under the cumulative ranking curve (SUCRA) was used to estimate the comparative efficacy of treatment regimens” is interesting and may be unfamiliar to many readers, including myself as reviewer.

It seems there is some inference with this method, especially were no direct comparison is made in the included studies.

If methods employed are sound (as assessed by someone knowledgeable in NMA) then I think this study has utility of suggesting further assessment of using adjunct G-CSF in severe melioidosis therapy and further well-planned prospective studies to assess the best eradication therapy.

I have no major concerns (assuming the analysis is performed correctly and is a valid application) but please see general comments, along with minor comments for revision.

***General comments:***

1. All the studies were from Thailand (line 246), this seems a bit unusual given your search strategy did not specify studies had to be a certain geographic area? Any comments on this and how it might affect your results/ implications/ conclusions? Given specific treatment modalities / local Thai strains or other factors.

**Response:** Our search terms, search strategies, and criteria for inclusion did not limit the location or country of the study in any way. All of the RCTs included in the Cochrane review were also conducted in Thailand, and our search terms can identify all of them. Since Thailand is a country with a high endemicity of melioidosis, we assume that all RCTs that evaluated the effectiveness of melioidosis therapies were carried out there. This could have an impact on how broadly applicable our study's findings are. We have mentioned this point in the limitation part (please see page 29, lines 558-560).

2. What are the current antibiotic guidelines for treating melioidosis in Thailand and do these differ from other country guidelines? Is G-CSF recommended in any Thailand treatment guidelines for melioidosis?

**Response:** The current guideline for treating melioidosis in Thailand has recommended ceftazidime, meropenem, or imipenem as the first-line treatment (1). This recommendation is similar to the treatment guideline in Australia (2) (please see the references below). G-CSF is not recommended for the treatment of severe melioidosis in Thailand due to the high cost of the drug and the non-significant benefit over ceftazidime suggested by the results of the RCT. We have discussed this point in the discussion part (please see page 25, line 457-459 and page 26, lines 469-473).

***References***

1.	Ministry of Public Health, D. o. C. D. (2021). "Guideline of Melioidosis." Retrieved 6th March, 2023, from http://klb.ddc.moph.go.th/dataentry/handbook/form/129.

2. Pitman, M. C., et al. (2015). "Intravenous Therapy Duration and Outcomes in Melioidosis: A New Treatment Paradigm." PLOS Neglected Tropical Diseases 9(3): e0003586.

3. Much of the discussion seems to focus a lot on comparison to Chloramphenicol/Doxy/TMP-STX to CAZ + G-CSF, however this seems a bit misplaced as this “triple therapy” should be rarely used now and is not really standard of care – I think the focus should be more on is CAZ + G-CSF better then monotherapy with CAZ or a carbapenem. In general, as a clinician many of the comparison are not overly useful.

**Response:** We have revised the summary of the results in the discussion part by emphasizing the highest risk of mortality rate in the chloramphenicol/Doxy/TMP-STX regimen (please see page 25, lines 444-447). In addition, our discussion has focused more on other treatment regimens (i.e., ceftazidime plus G-CSF, ceftazidime plus TMP-SMX, and carbapenem) according to your comments (please see page 26-27).

4. Is it worth outlining a bit more what SUCRA values actually mean to people who are not familiar with NMA? Its easier for most readers to understand what RR are whether this is significant or not – however much of your results rely on the SUCRA values, when for example CAZ vs CAZ + G-CSF is not significant, RR 0.81 (0.61-1.06). But then your SUCRA for CAZ + G-CSF is 79.7% (the highest) and therefore the conclusion is this is most likely the best therapy. I think it could be discussed contextualised a bit better how you have come to this conclusion.

**Response:** The meaning of SUCRA values is more fully explained in the statistical analysis part (please see page 10, lines 215–220). As per your and other reviewers' comments, we have revised the conclusion in the main text, abstract, and author summary and also revised the results in the abstract (please see page 2, lines 34-37).

***Minor:***

1. Line 31: Full stop/ period after regimens.

**Response:** Already corrected (page 2, line 31).

2. Line 50: Many guidelines suggest meropenem, any reason to state imipenem instead?

**Response:** We have corrected this sentence (please see page 4, line 55).

3. Line 70: Small distinction, but it is the U.S. Centers [with an s] of Disease…

**Response:** We have corrected this error (please see page 5, line 80).

4. Line 74: Suggest delete “continuously”

**Response:** This word has been deleted (please see page 5, line 84).

5. Line 76: “But the death rate doubly increases when patients have septicemia”- rewrite without the phase “doubly increases” Note also ref 5 has had a retraction due to significant issues with the article – consider not using this reference. An issue I noted, for example, was was that the 21% mortality rate stated (in Thailand) had a reference to a case report of glanders in a human, or is not referenced when this same rate is stated to be the mortality rate in Australia.

**Response:** This paragraph has been revised according to your comment by stating the range of mortality rate instead of quoting only a single value (please see page 5, lines 85-88)

6. Line 77: Carbapenem is not an antibiotic as such, rather a class of antibiotics

**Response:** Due to the comments below, we have revised the content of this paragraph and deleted this word (please see page 5, lines 88-90).

7. Line 78-79: “Mortality rate was still high (37%), especially in patients with septicemia” This might need a bit more context as you state antibiotics reduced mortality rate but in a previous sentence say the mortality rate is approx 41% with reference to another study. Is this comparing untreated/ undertreated to optimally treated etc?

**Response:** We have revised this paragraph according to your comments (please see page 5, line 91).

8. Line 83-84: “Unfortunately, none of these studies demonstrated significant benefit of these treatment regimens over ceftazidime to reduce mortality in severe melioidosis patients”- were all these studies comparing a treatment regimen to ceftazidime?

**Response:** All except one study (Thamprajamchit et al) compared treatment regimen to ceftazidime. The reference of Thamprajamchit et al has been deleted from this sentence (please see page 5, line 95).

9. Line 132-134: Often the reviewers who did the review for selection and the 3rd party consensus would have this defined by bracketed initials.

**Response:** The initials of reviewers and 3^rd^ party have been defined (please see page 8, lines 160 and 163).

10. Line 154: What do you mean by “clinical-confirmed” recurrence?

**Response:** Clinical-confirmed was defined as the development of new symptoms and signs of infection that were consistent with melioidosis, but in the absence of a positive *B. pseudomallei* culture from any site. We have stated this definition in the main text (please see page 9, lines 185-187).

11. Line 155: Typo- Clulture

**Response:** Already corrected (page 9, line 183).

12. Line 164: As above which reviewer initials?

**Response:** Reviewer initials have been cited (please see page 9, line 194-195).

13. Line 164-166: “Outcomes of interest including frequency of patients who developed or did not develop the outcomes for each treatment were also extracted”- rewrite.

**Response:** This sentence has been rewritten (please see page 9, line 195-196).

14. Line 176-178: As per prior comments consider specifying which reviewers did what.

**Response:** Reviewer initials have been specified (please see page 10, line 206)

15. Line 201-202 and Fig 1, line 229-235: It is a bit confusing from reading the text and comparing to this figure- did you have 8 or 9 studies that underwent analysis for treatment of severe melioidosis?

**Response:** Eight studies were included for treatment of severe melioidosis. However, one study (Chierakul et al) had two sub-trials with different study’s population. Therefore, we considered these two sub-trials as the different studies in the meta-analysis. Thus, 9 studies underwent analysis for treatment of severe melioidosis. We have revised Figure 1 to make them correspond (please see page 11, Figure 1).

16. Line 247-249: Make it clear you are also not just looking at a catch all “co-morbid conditions” “The rates of the co-morbid conditions of diabetes mellitus and chronic kidney disease ranged..”

**Response:** We have revised this sentence to make it clearer (please see page 12, lines 284-286).

17. Line 250: Keep consistent decimal points.

**Response:** This number has been corrected (please see page 12, line 287).

18. Line 260, Tab 1 Sookpranee, 1992: Pneumonia box is empty.

**Response:** This study did not report the number of patients who had pneumonia. We have stated NR (not reported) in the Table 1.

19. Line 267-273: “Treating with chloramphenicol plus doxycycline plus TMP-SMX had a significantly greater risk of hospital mortality than other treatments except for cefoperazone-salbactam plus TMP-SMX, with RRs of 2.22 (95% CI: 1.28-3.86) for ceftazidime plus TMP-SMX, 1.96 (95% CI: 1.13-3.38) for amoxicillin-clavulanic acid, 2.01 (95% CI: 1.12-3.59) for Imipenem, 2.38 (95% CI:1.39-4.07) for ceftazidime plus G-CSF, and 1.92 (95% CI: 1.21-3.05) for ceftazidime. Ceftazidime plus G-CSF had a lower hospital mortality rate than all other treatments, although the results were not statistically significant”

Where did you get the RR of 2.22 from- this is not in the table?

I take it you single out the treatment regimen of chloramphenicol/ doxycycline/ TMP-SMX because this had the most significant results compared to other treatments but is this clinically relevant – is this regimen used much in practice? It might be worth highlighting some of the RR, even if not significant for other treatment regimens that are often used.

**Response:** Since the results (risk ratios of treatment comparisons) in Table 2 are read from right to left. The RR of ceftazidime+TMP-SMX compared to TMP-SMX+doxycycline+chloramphenicol is 0.45 (95% CI: 0.26-0.78). Thus the RR of TMP-SMX+doxycycline+chloramphenicol versus ceftazidime+TMP-SMX is the inverse of 0.45 that is equal to 2.22. So we included this number (2.22) in the main text.

We have highlighted some RRs of imipenem and ceftazidime+TMP-SMX which are the treatment regimens recommended in the guideline in the main text as you suggested (please see page 14, lines 310-314).

20. Line 280-297, Tab 2: A somewhat busy and hard to interpret table. This might be my unfamiliarity with NMA, but clearly comparisons are made between treatment regimens such as imipenem vs co-amoxyclav or TMP-STX + doxy + chloramphenicol vs imipenem (which is a significant result). Is might be worth outlining which results are “derived” or indirect vs from direct comparison from studies (which would seem to hold more weight).

**Response:** We have outlined which results are derived from direct comparison only (blue highlighted), from both direct and indirect comparisons (yellow highlighted), and from indirect comparison only (green highlighted), as you suggested (see Table 2, 4, and 5).

21. Line 293: “disseminated disease”- how is this defined? Compared to bacteraemia.

**Response:** Disseminated disease was defined as a positive blood culture result plus >1 noncontiguous focus of infection. We have specified this definition in the main text and in Table 3 (please see page 16, lines 337 and page 18, line 343).

22. Line 318-323, Tab 4: Similar to comments on table 2. Consider outlining direct comparison vs NMA derived values.

**Response:** We have outlined which results are derived from direct comparison only (blue highlighted), from both direct and indirect comparisons (yellow highlighted), and from indirect comparison only (green highlighted), as you suggested (see Table 2, 4, and 5).

23. Line 371-376 Tab 5, comments per other tables.

**Response:** We have outlined which results are derived from direct comparison only (blue highlighted), from both direct and indirect comparisons (yellow highlighted), and from indirect comparison only (green highlighted), as you suggested (see Table 2, 4, and 5).

24. Line 403-404: “Currently, ceftazidime with or without TMP-SMX is the preferred first line treatment for severe melioidosis”- reference any relevant guidelines. Define specifically when addition of TMP-SMX is thought to be of benefit – i.e. patients with focal disease rather than just pneumonia/ bacteraemia.

**Response:** We have revised this paragraph according to the previous comments and the relevant guidelines have been cited (please see page 25, lines 454-455). The indications for combining TMP-SMX with ceftazidime has been discussed and stated according to the treatment guideline developed by the Darwin group (please see page 26, lines 480-486).

25. Line 478-479: “Inclusion of more data from more studies would strengthen our conclusion”. Well, it might not – I think you need to highlight that your findings are perhaps more hypothesis generating, far from good quality evidence to suggest G-CSF should definitively be utilised and that we need well designed prospective studies looking into therapy for melioidosis. I think the fact that direct meta-analysis could not be performed on “standard therapies” for melioidosis and NMA had to be performed – with many results lacking power / not reaching significance- really highlights the need for good quality clinical trials to improve treatment of melioidosis.

**Response:** We have revised the conclusions in main text, abstract, and author summary to tone down the suggestion of using G-CSF for treatment of severe meiloidosis. In addition, the non-significant results of ceftazidime plus G-CSF and requirement of additional good quality clinical trials have been emphasized according to your suggestions (please see page 29, lines 562-570).
