# Supplementary material for: Efficacy of drug treatment for severe melioidosis and eradication treatment of melioidosis: A systematic review and network meta-analysis
Source: PLoS Negl Trop Dis. 2023 Jun 12;17(6):e0011382. doi: 10.1371/journal.pntd.0011382 (PMC10289671; doi:10.1371/journal.pntd.0011382)
Supplement: Attachment — Submitted filename: response.docx [file pntd.0011382.s020.docx]

**Editor’s comments**

1. The editor respectfully requests that the manuscript be reviewed from clarity and grammar before it can be accepted for publication.

**Response:** The manuscript has been reviewed and edited by a native English speaker.

**Reviewer’s comments**

Reviewer's Responses to Questions

<b>Key Review Criteria Required for Acceptance?</b></br></br> As you describe the new analyses required for acceptance, please consider the following:</br></br>

<b>Methods</b></br></br>

-Are the objectives of the study clearly articulated with a clear testable hypothesis stated?</br> -Is the study design appropriate to address the stated objectives?</br> -Is the population clearly described and appropriate for the hypothesis being tested?</br> -Is the sample size sufficient to ensure adequate power to address the hypothesis being tested?</br> -Were correct statistical analysis used to support conclusions?</br> -Are there concerns about ethical or regulatory requirements being met?</br></br>

**Reviewer #1:** The author addressed a clear objective in the study, and the design used to prove the hypothesis could be considered appropriate.

**Response:** Thank you very much for your suggestions for improving the quality of our manuscript.

--------------------

<b>Results</b></br></br>

-Does the analysis presented match the analysis plan?</br> -Are the results clearly and completely presented?</br> -Are the figures (Tables, Images) of sufficient quality for clarity?</br></br>

**Reviewer #1:** The study results were completely presented, and the figures were easy to understand.

**Response:** Thank you very much for your suggestions for improving the quality of our manuscript.

--------------------

<b>Conclusions</b></br></br>

-Are the conclusions supported by the data presented?</br> -Are the limitations of analysis clearly described?</br> -Do the authors discuss how these data can be helpful to advance our understanding of the topic under study?</br> -Is public health relevance addressed?</br></br>

**Reviewer #1**: After revision, the conclusions are clearer, and the chance of misleading the reader is less than in the previous version.

**Response:** Thank you very much for your suggestions for improving the quality of our manuscript.

--------------------

<b>Editorial and Data Presentation Modifications?</b></br><br/> Use this section for editorial suggestions as well as relatively minor modifications of existing data that would enhance clarity. If the only modifications needed are minor and/or editorial, you may wish to recommend “Minor Revision” or “Accept”.

**Reviewer #1:** The author completely addressed my concern, and I have no further corrections.

**Response:** Thank you very much for your suggestions for improving the quality of our manuscript.

**Reviewer #3:** Needs just minor revision of grammar / spelling of edited sections. Also needs consistent spacing of references - sometimes references[1] are immediately after and sometimes spaced [1] as demonstrated.

**Response:** The manuscript has been reviewed and edited by a native English speaker. We have revised the manuscript for consistent spacing of references.

--------------------

<b>Summary and General Comments</b></br></br> Use this section to provide overall comments, discuss strengths/weaknesses of the study, novelty, significance, general execution and scholarship. You may also include additional comments for the author, including concerns about dual publication, research ethics, or publication ethics. If requesting major revision, please articulate the new experiments that are needed.

**Reviewer #1:** The treatment of melioidosis remains a significant issue in endemic areas. This study provides valuable data from numerous landmark trials for clinicians to select the most suitable regimens for their patients. The thoroughness of the data is a strength of the study.

As for my concerns from previous versions, applying network meta-analysis (NMA) may not lead to a conclusive answer to the author's question. Therefore, readers must carefully consider their interpretation.

**Response:** In the previous version, we revised our conclusions in the abstract, author's summary, and main text and we discussed the validity of our NMA in the discussion and the limitation sections according to the reviewer's comments.

**Reviewer #2:** I am reasonably happy that the authors have now included sufficient caveats to their conclusions to address my original concerns. However, despite the assurance that the English has been reviewed by a native speaker I did find a number of instances where I felt it could be improved, particularly in the new text, much of which reads very much as though it has been written by a non-native and some of which is a little difficult to understand. I have included a few specific points below but I would suggest that the whole paper should be reviewed again by the native speaker, paying particular attention to the new text.

**Response:** The manuscript has been reviewed and edited by a native English speaker.

Additionally, altough the authors have attempted to address my comment about the reference to the WHO Biosafety Manual, they now inapproporiately suggest that the 2020 (4th) edition of the WHO Laboratory Biosafety Manual suggests that B. pseudomallei is "considered a risk group 3 pathogen by the World Health Organization (WHO)". In fact this edition goes to some lengths to say:

"Regardless of the approach used, the classification of biological agents and/or the work being performed with them should not be considered static, nor should it be universally applied across jurisdictions. Classification can vary according to contextual factors (for example, geography, time, process), so the application of one country’s classification system to another country should be avoided as it could create confusion and result in inadequate or excessive risk control measures".

**Response:** We have removed this sentence from the manuscript and the reference has been updated (please see page 5, lines 79-81).

Finally, reference 8 persists - it has been withdrawn and should not be cited.

**Response.** This reference has been deleted from the manuscript (please see page 5).

Examples of English issues (lines cited are from the revised Word version)

- L 91. Add 'the' before 'mortality rate'.

**Response:** Already added (please see page 5, line 91).

- L 185. Recurrence misspelled. In addition I would suggest using 'clinically suspected' rather than 'clinical confirmed'.

**Response:** This term has been revised according to your suggestion (please see page 9, lines 183-185).

- L 225 and elsewhere. The spaces between the text and the references have been lost.

**Response:** We have revised the manuscript for consistent spacing of references.

- LL 283 and 286. Use 'studies'' (plural) not 'study's' (singular).

**Response:** Revised.

- L 311-2. I would say 'failed to achieve staistical significance'.

**Response:** This sentence has been revised according to your suggestion (please see page 14, line 312).

- L 312. Suggest replacing 'reduced' with 'lower'.

**Response:** This sentence has been revised according to your comment (please see page 14, line 313).

- L 448-9. Rewrite.

**Response:** This paragraph has been edited by a native English speaker (please see page 25, lines 449-451).

- L 473-5. Rewrite.

**Response:** This paragraph has been edited by a native English speaker (please see page 26, lines 473-477).

- L 496-509. Review and rewrite.

**Response:** This paragraph has been reviewed and edited by a native English speaker (please see page 27, lines 497-509).

- L 522-529. Review and rewrite.

**Response:** This paragraph has been reviewed and edited by a native English speaker (please see page 28, lines 523-529).

- L 544-553. Review and rewrite.

**Response:** This paragraph has been reviewed and edited by a native English speaker (please see page 29, lines 546-562).

- L 558. Rewrite

**Response:** This paragraph has been reviewed and edited by a native English speaker (please see page 29, lines 562-565).

- L 562. Explain what you mean.

**Response:** This statement has been revised to make it more comprehensible (please see page 29, lines 567-570)

Reviewer #3: I think the authors have done a good job of responding to reviewer comments.

**Response:** Thank you so much for your suggestions for improving the quality of our manuscript.
